# CRISPR/Cas9 genome editing of potato *St*DMR6-1 results in plants less affected by different stress conditions

**DOI:** 10.1093/hr/uhae130

**Published:** 2024-05-06

**Authors:** Milla Karlsson, Nam Phuong Kieu, Marit Lenman, Salla Marttila, Svante Resjö, Muhammad Awais Zahid, Erik Andreasson

**Affiliations:** Department of Plant Protection Biology, Swedish University of Agricultural Sciences, Box 190, 234 22, Lomma, Sweden; Department of Plant Protection Biology, Swedish University of Agricultural Sciences, Box 190, 234 22, Lomma, Sweden; Department of Plant Protection Biology, Swedish University of Agricultural Sciences, Box 190, 234 22, Lomma, Sweden; Department of Plant Protection Biology, Swedish University of Agricultural Sciences, Box 190, 234 22, Lomma, Sweden; Department of Plant Protection Biology, Swedish University of Agricultural Sciences, Box 190, 234 22, Lomma, Sweden; Department of Plant Protection Biology, Swedish University of Agricultural Sciences, Box 190, 234 22, Lomma, Sweden; Department of Plant Protection Biology, Swedish University of Agricultural Sciences, Box 190, 234 22, Lomma, Sweden

## Abstract

Potato is the third most important food crop, but cultivation is challenged by numerous diseases and adverse abiotic conditions. To combat diseases, frequent fungicide application is common. Knocking out susceptibility genes by genome editing could be a durable option to increase resistance. *DMR6* has been described as a susceptibility gene in several crops, based on data that indicates increased resistance upon interruption of the gene function. In potato, *Stdmr6-1* mutants have been described to have increased resistance against the late blight pathogen *Phytophthora infestans* in controlled conditions. Here, we present field evaluations of CRISPR/Cas9 mutants, in a location with a complex population of *P. infestans*, during four consecutive years that indicate increased resistance to late blight without any trade-off in terms of yield penalty or tuber quality. Furthermore, studies of potato tubers from the field trials indicated increased resistance to common scab, and the mutant lines exhibit increased resistance to early blight pathogen *Alternaria solani* in controlled conditions. Early blight and common scab are problematic targets in potato resistance breeding, as resistance genes are very scarce. The described broad-spectrum resistance of *Stdmr6-1* mutants may further extend to some abiotic stress conditions. In controlled experiments of either drought simulation or salinity, *Stdmr6-1* mutant plants are less affected than the background cultivar. Together, these results demonstrate the prospect of the *Stdmr6-1* mutants as a useful tool in future sustainable potato cultivation without any apparent trade-offs.

## Introduction

Current challenges for agri- and horticultural production systems include disease management, the transition towards sustainable practices, and adaptation to the effects of climate change, which likely will include increased drought and salinity of soils [1, 2]. At the same time, global population is projected to continue rising throughout this century and increasing food demands need to be fulfilled [3].

Potato (*Solanum tuberosum L.*) is a widespread staple crop and the third most important food crop in the world (https://www.fao.org/faostat/). Potato tubers are a good source of nutrients, such as carbohydrates, proteins, minerals, and vitamin C [4, 5], and produce a higher yield per hectare than any of the other top food crops wheat, rice or maize (https://www.fao.org/faostat/), which makes it a candidate for providing calories and nutrients where there are deficiencies [4–6]. However, potato cultivation is challenged by a number of diseases and pests, such as blights, viral diseases, scabs, and cyst nematodes (https://cipotato.org/potato/potato-pests-diseases/). The late blight disease, caused by the oomycete pathogen *Phytophthora infestans*, is a main target in resistance breeding but is still largely controlled by repeated fungicide application, which is not regarded as a sustainable practice. Resistance genes (R-genes) are generally scarce and are frequently overcome by pathogen evolution, rendering them ineffective when deployed individually [7]. Single or no R-genes are currently available for other important diseases, such as the foliar disease early blight caused by *Alternaria solani* that impacts yield and currently also requires fungicide application, and tuber skin scabs that affect marketability.

Susceptibility genes (S-genes) that are exploited by pathogens to facilitate their survival and proliferation in the host, could be functionally excluded from the genome to increase resistance [8]. In recent years, there has been growing interest in the use of S-gene knock-outs in potato research, particularly for their potential to reduce susceptibility to late blight [9–13]. However, an S-gene knock-out may also confer broad-spectrum resistance, as these types of genes often suppress general defence responses [8]. In *Arabidopsis thaliana*, the S-gene *DOWNY MILDEW RESISTANT 6* (*AtDMR6)* was shown to encode a salicylic acid 5-hydroxylase (S5H) in a major catabolizing pathway of salicylic acid (SA), a phytohormone involved in defence responses [14, 15]. Recently, the potential of deleting *DMR6* to increase resistance has been explored in several crops, and increased resistance has been found, e.g. to hemibiotrophic bacteria and biotrophic fungi in the close potato relative tomato, hemibiotrophic bacteria in banana, a biotrophic oomycete in sweet basil, hemibiotrophic bacteria in citrus, a biotrophic oomycete in grapevine, to hemibiotrophic bacteria and fungi in rice and to hemibiotrophic fungi in barley [16–22]. Most commonly, the DMR6 (S-gene) system has demonstrated effectiveness in reducing downy mildew disease, which originally earned it the name [23]. Until now, no resistance has been indicated towards necrotrophic pathogens.

In potato, the function of *St*DMR6-1 has been distinguished from *St*DMR6-2, as the *Stdmr6-1* knock-out showed increased resistance to *P. infestans*, while *Stdmr6-2* did not [24]. However, increased late blight resistance of the *Stdmr6-1* potatoes has only been evaluated in controlled conditions.

SA is involved in regulation of a diverse range of physiological processes that include regulation of growth and development but it is mainly associated with defence responses [25]. The expression of SA-induced genes increase in *Stdmr6*-silenced potato plants exposed to *P. infestans* infection [12]. Silencing of the corresponding *S5H* genes in rice was shown to increase the intrinsic level of SA in the plant tissue, and increased broad-spectrum disease resistance in the crop [21]. Similarly, broad-spectrum resistance was shown in *Sldmr6-1* tomatoes (*Solanum lycopersicum*) [19]. However, broad-spectrum biotic resistance by *dmr6* mutation has not been described before in potato or any other tuber or root crop.

In addition to biotic stress regulation, SA has been associated with abiotic stress responses, e.g. in conditions such as salinity or drought [26–29]. Foliar application of SA on potato can alleviate stress caused by salinity by increasing antioxidant activity and osmolytes, improving water relations, gaseous exchange, morphological parameters, tuber yield, and K^+^ contents, although the SA concentration could be an important factor [30]. Increased resistance to abiotic stress has not been indicated in any *dmr6* plants.

**Figure 1:**
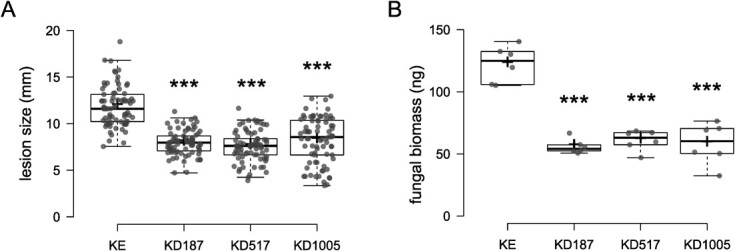
Disease quantification 5 days post inoculation by *A. solani* on leaves in whole plant assays. KE denotes the cultivar King Edward background, and KD denotes the Stdmr6-1 mutant lines. Asterisks denote significant difference as compared to KE (^***^P < 0.001). (A) Lesion sizes, n = 77. (B) Relative fungal biomass per mg sample, n = 6.

Because of the broad involvement of SA in defence responses, we hypothesized that the *Stdmr6-1* knock-out could aid plant vigor both under infection by diverse pathogens and under abiotic stress conditions. Furthermore, we conducted four years of field trials where data has been collected regarding field resistance to *P. infestans.* Tuber quality and yield were also analysed.

## Results and discussion

The aim was to investigate the presence of broad-spectrum resistance, encompassing both biotic and abiotic stressors, in the *Stdmr6-1* mutant plants. Most of these experiments were conducted under controlled conditions, which are presented in the first section of the results. Additionally, a series of quality tests were performed on tubers obtained from field trials. To assess resistance to late blight, which had been previously documented only under controlled conditions with a single strain of *P. infestans*, spontaneous infection was monitored during four years of field trials. Analysis of field trial data encompasses the second section of the results. 

### Experiments under controlled conditions

#### Early blight infection assay

Previously, we described increased resistance to the hemi-biotrophic pathogen *P. infestans* in the *Stdmr6-1* mutants (denoted as KD lines) [24]. It was of interest to additionally test resistance against an agriculturally important necrotrophic pathogen. *Alternaria solani*, the necrotrophic fungus responsible for the globally important foliar disease early blight, was used for this purpose. The leaves of five weeks old plants were infected in a whole plant assay, and subsequent lesion development was measured at five dpi as lesion diameter (Fig. 1A). All *Stdmr6-1* lines exhibited smaller lesion sizes, indicating greater resistance against the necrotrophic fungal pathogen *A. solani* and suppression of its growth. This conclusion is also supported by qPCR measurement of pathogen DNA abundance transformed to pathogen biomass (Fig. 1B). The visible symptoms and the pathogen biomass ratio followed a similar trend, with significantly lower levels of early blight in all mutant lines compared to the King Edward (KE) background.

In the model plant *A. thaliana*, it has been suggested that plant defence involving SA is primarily linked to defence against biotrophs and hemi-biotrophs, while jasmonic acid (JA) is the dominant hormone regulating defence against necrotrophs [31]. However, it has been shown that SA signalling is required in potato for defence against the nectrotroph *A. solani*, indicating that the relationships between the mentioned phytohormones and defence might be different in potato [32]. Now, we show that removal of an SA catabolic gene decreases symptoms of this pathogen. More specifically, it is the first time a *dmr6* mutant of any crop is shown to have increased resistance to a necrotrophic pathogen.

#### Common scab resistance in stored potato tubers

**Figure 2:**
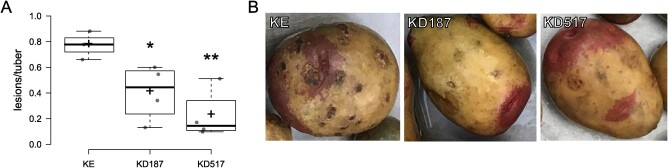
Common scab on potato tubers. (A) The average number of lesions per tuber in one field plot represents one replicate. Asterisks denote significant difference as compared to KE (^*^P < 0.05, ^**^P < 0.01, n = 3–4). (B) Pictures of tubers from the KE line, KD187 line, and KD517 line. The common scab lesions can be seen covering parts of the KE tuber, and a lesser number of lesions are visible on the KD187 and KD517 tubers.

Bacterial scabs on potato are caused by species in the *Streptomyces* genus of Actinobacteria, which often are present in soils [33]. The pathogen was present at the site of our multi-year field trial in southern Sweden, causing spontaneous infection and symptoms on tubers. Quantification of scab lesions on harvested tubers that had been stored in a cold room showed a significantly lower severity of the disease in each of the mutant lines as compared to KE background (Fig. 2A). Representative tubers from KE and the mutant lines can be seen in Fig. 2B.

While development of skin lesions can lead to secondary infections, potato skin scabs such as common scab mostly affect the marketability of tubers and do not primarily affect yield. Impact on marketability, however, leads to food waste. Prevention of common scab disease is usually done by crop rotation and by the use of certified seed tubers, but is complicated due to the commonplace presence of pathogens in soils [33]. Resistance has been suggested as the best practice of combating the disease, but resistant cultivars are scarce [33]. The broad-spectrum resistance in the *Stdmr6-1* potato could thereby aid towards decreasing the severity of common scab in potato.

#### Salt stress

Considering the indications of an increased broad-spectrum biotic stress resistance, and the involvement of salicylic acid in both biotic and abiotic stress responses, it was of interest to investigate whether the increased resistance extended to plant tolerance of abiotic stress. Soil salinization poses a common and detrimental abiotic challenge to agricultural areas, and crops that are tolerant to growing in such conditions could be part of a solution for how to address these areas.

**Figure 3:**
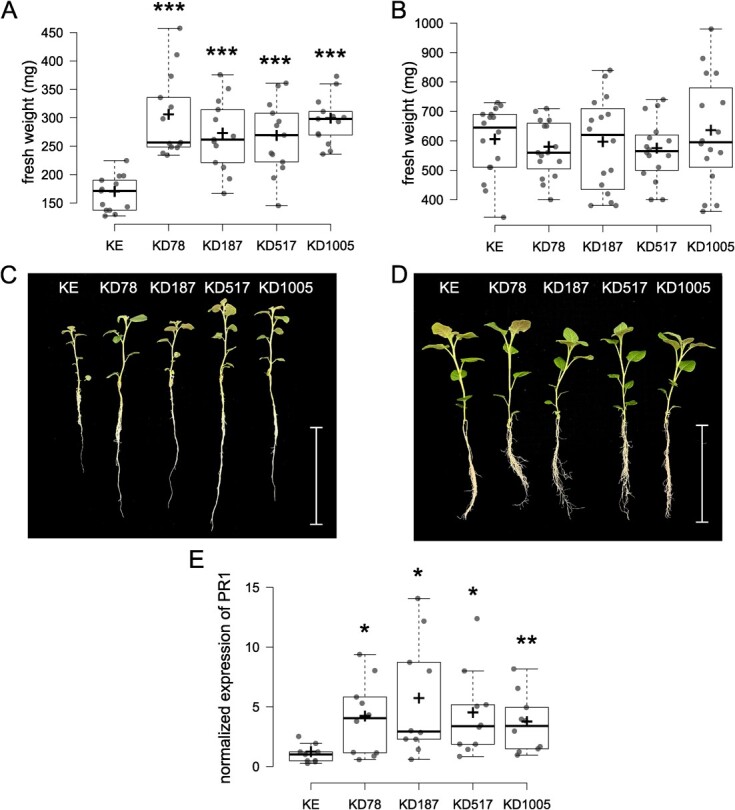
Results of growth experiment in salinity. KE denotes the cultivar King Edward background, and KD denotes the *Stdmr6-1* mutant lines. Asterisks denote significant difference as compared to KE (^*^P < 0.05, ^**^P < 0.01, ^***^P < 0.001). (A) Fresh weight in mg of KE and four *Stdmr6-1* mutant lines after exposure to 60 mM NaCl for 7 days, n = 13. (B) Fresh weight in mg of plants grown in the control conditions without added NaCl, n = 16. (C) Relative size of one representative plant from each line after the experiment with 60 mM NaCl. The scale bar represents 100 mm. (D) Relative size of one representative plant from each line in the control experiment without added NaCl. The scale bar represents 50 mm. (E) Normalized expression of SA marker gene *PR1* in samples collected 12 hours after initiation of the salt treatment.

Tolerance to NaCl was assessed in young *Stdmr6-1* plants using a hydroponic system and was quantified by measuring fresh weight after one week of growth under the saline conditions. Each of the mutant lines had grown to significantly larger plants than KE (background), as quantified by a heavier fresh weight (Fig. 3A). Representative plants of each line can be seen in Fig. 3C. In growth experiments without salt, no weight difference was seen among any of the lines or the background genotype (Fig. 3B). Representative plants of each line from the control group can be seen in Fig. 3D. Additionally, the abundance of the SA marker gene *PR1* in samples collected 12 hours after initiation of the salt treatment was analysed (Fig. 3E). The function of *Stdmr6-1* was confirmed by significantly higher abundance of PR1 in all mutant lines, while no difference was seen in untreated samples (not shown). Resistance in *St*DMR6-silenced plants has previously been associated with a super induction of SA-mediated signalling pathways during infection by *P. infestans* [12], which is consistent with our findings. However, the measured PR1 levels under salt stress are not as striking as during pathogen infection.

Growth of the mutant lines was impaired by the salt treatment compared to control conditions without salt stress, but to a lesser extent than the growth of KE. This suggests that the *Stdmr6-1* mutants exhibit greater tolerance to saline conditions and maintain a higher growth rate compared to the background genotype. This indication of improved growth could be compared to the improved morphological parameters, and even yield, found after foliar application of SA to potato plants under salt stress [30]. Similar results of improved growth have also been presented regarding other important crops, such as maize and rice under salt stress [34, 35]. Various horticultural crops have shown improved tolerance to stresses, including salinity, osmotic stress, heat- and cold-stress, heavy metals, and radiation upon SA supplementation, as reviewed by Chen *et al.* [26]. However, the intrinsic modification of SA regulation by *dmr6-1* mutation would be easier to handle in large-scale growth conditions, compared to the more extensively studied method of extrinsic application of the hormone or derivates thereof. However, to confirm our results and determine if *Stdmr6-1* could be a viable option for resilient cultivation in saline soil, it is essential to conduct field trials in such circumstances.

#### Drought simulation experiments

Drought affecting agricultural land is a prevalent environmental concern. After indications of abiotic stress tolerance in salt stress experiments, the *Stdmr6-1* plants were therefore further subjected to experiments of mimicking controlled drought by imposing osmotic stress in hydroponics, and in short-term drought experiments in soil.

**Figure 4:**
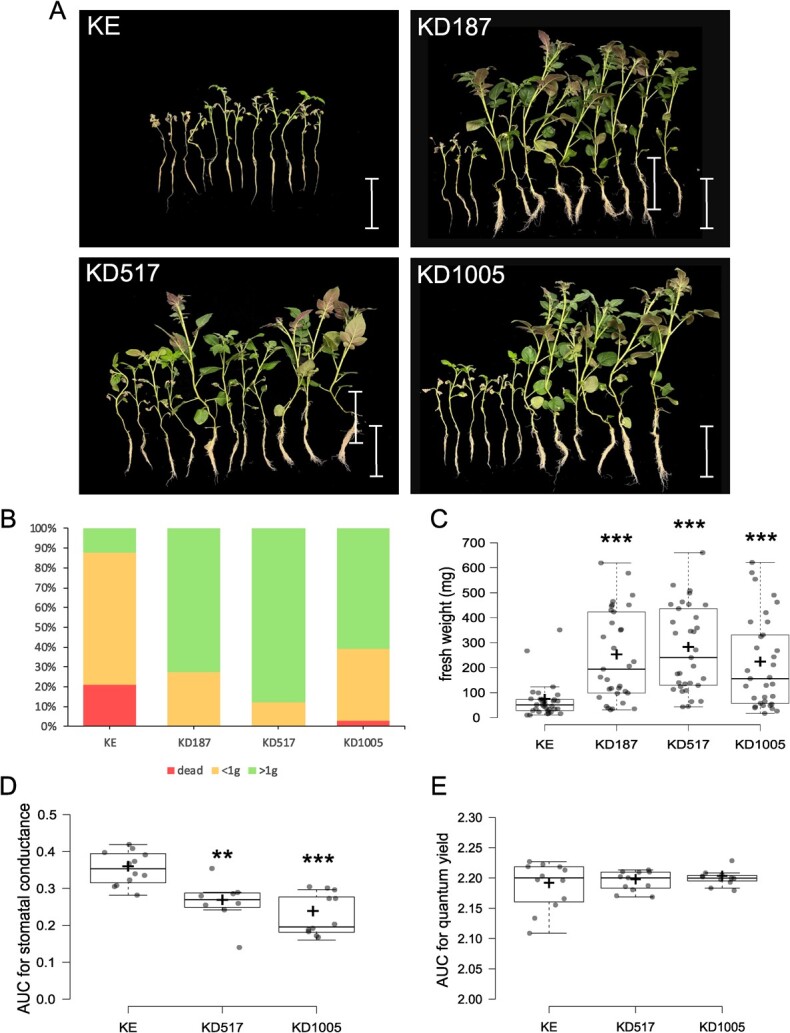
Drought simulation experiments by PEG treatments and in soil. (A) Pictures of recovery phenotypes two weeks after exposure to PEG-6000 are given for lines KE (cultivar King Edward background), KD187, KD517, and KD1005. The scale bars represent 100 mm. (B) The distribution in percentage of plants that died, recovered to a fresh weight of below one gram, or recovered to a fresh weight of above one gram 2 weeks after exposure to PEG-6000, n = 33. (C) Fresh weight of all plants after two weeks recovery post exposure to PEG-6000, n = 33. (D) Area under curve (AUC) for stomatal conductance (mol∙m^−2^∙s^−1^) days seven through ten of drought experiments in soil, n = 8–12. (E) AUC for quantum yield of Photosystem II (ΔF/Fm′) Days 7 through 10 of drought experiments in soil, n = 11–12.

In the initial experiments involving imposed osmotic stress, the plants were subjected to a hydroponic system for two weeks before being deprived of water uptake by replacing the growth medium with a 20% PEG-6000 solution. After 24 hours, the solution was removed and plants were re-cultured and left to recover and grow for two additional weeks. Fresh weight was then measured of individual plants in each line and survival rate was noted (Fig. 4A, B). The mutant lines had generally grown significantly larger during the recovery time, compared to KE (background) (Fig. 4C). The fraction of dead plants was 21% for KE, 3% for KD1005 and 0% for the other mutant lines. Results are from three iterations of the experiment combined, each with similar results.

In the second set of experiments, plants were potted in soil and kept under well-watered conditions, with soil moisture levels at 70% to 80% of the water capacity, for a period of two weeks. Subsequently, watering was withdrawn, and the soil water content was monitored daily, as well as stomatal conductance, and quantum yield of Photosystem II. A difference in the area under curve (AUC) for stomatal conductance during Days 7 to 10, was observed between KE and the mutant lines KD517 and KD1005, respectively (Fig. 4D). We could not observe any difference in quantum yield during this time (Fig. 4E). Plants were allowed to recover on Day 21 post watering by immersing the pots in water. The recovery rate was assessed two days later, and the fresh weight of above-ground tissues was measured. We could not find any significant differences in the recovery rate or fresh weight under our experimental conditions with relatively high humidity (Supplementary data S1).

In a study by Poor *et al.* [36], tomato plants treated with exogenous SA exhibited lower stomatal conductance during the first week, while it was restored in the longer term. Similarly, we saw a decrease of stomatal conductance in the initial stages of drought, starting while the soil water capacity reached around 40% on Day 7, down to around 20% on Day 10, levels at which a stress response could be expected to have been initiated. No difference in stomatal conductance was seen during the later stages of drought (data not shown). Stomatal closure-induced drought tolerance caused by endogenous build-up of SA has also been shown in Arabidopsis [37]. Hence, our data suggest that deletion of *StDMR6-1* allows faster adaptation to the condition of initial drought by regulation of stomata in the soil experiments. In the PEG-induced osmotic stress experiment, it is possible the tolerance to the sudden and severe stress might also have been aided by additional beneficial properties associated with elevated SA levels, such as osmolyte accumulation or ROS scavenging [29].

#### Tuber quality

Tuber characteristics are important for the marketability of a cultivar. Using tubers harvested from the field season 2023, tuber hardness was measured with a penetrometer, on each half of five halved raw tubers. Average force needed for KE was 109N, 107N for KD187, and 108N for KD517. Two-tailed Student's *t*-tests against KE (background) showed no significant difference (n = 10). This indicates that no major texture differences are present in tubers from the mutant lines.

**Figure 5:**
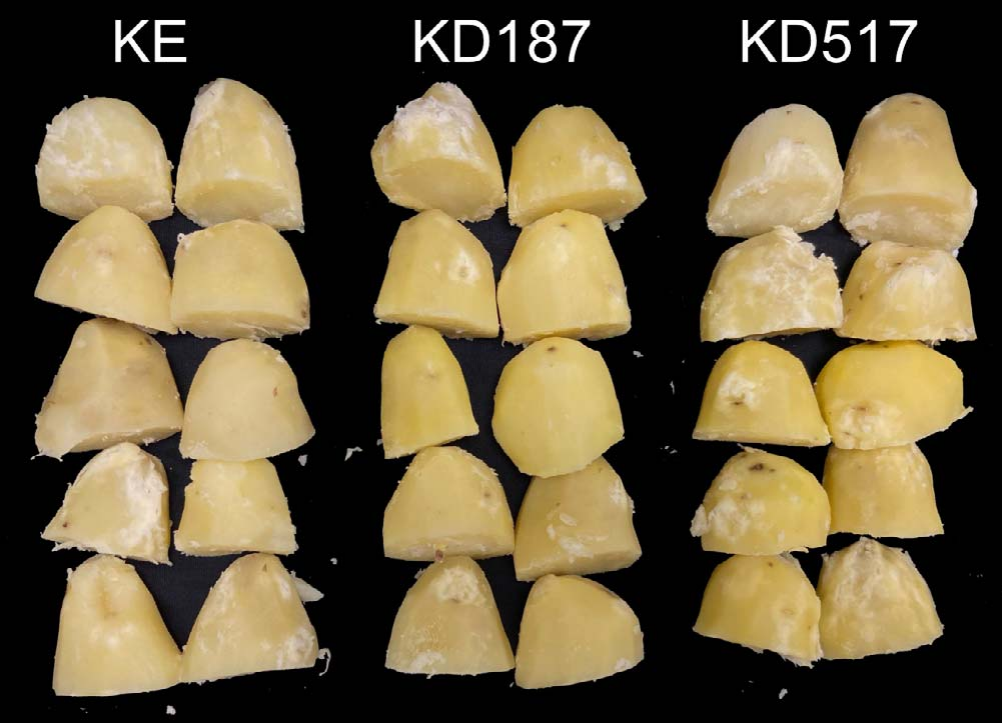
Cooking quality test of five tubers of each line from the field trial in 2023. KE denotes the cultivar King Edward background, and KD denotes the *Stdmr6-1* mutant lines.

Specific gravity was calculated for each of the genotypes, resulting in no significant difference as calculated in two-tailed Student's *t*-test against KE (background) (n = 5). Mean values for specific gravity were 1.10 for KE, 1.09 for KD187, and 1.10 for KD517. This indicated similar ratios of dry mass [38]. Dry mass ratio was further confirmed by weighing fresh and dried tubers. Average dry mass was 27.6% for KE (background), 28.9% for KD187, and 28.4% for KD517, which indicates high-quality tubers [39]. No significant difference was measured with two-tailed Student's *t*-tests (n = 5). Tubers were also used for a standard cooking quality test (Fig. 5). No obvious quality difference was observed during this test, as all tubers remained similarly whole and non-soggy.

### Data from field trials

#### Field resistance to late blight

**Figure 6:**
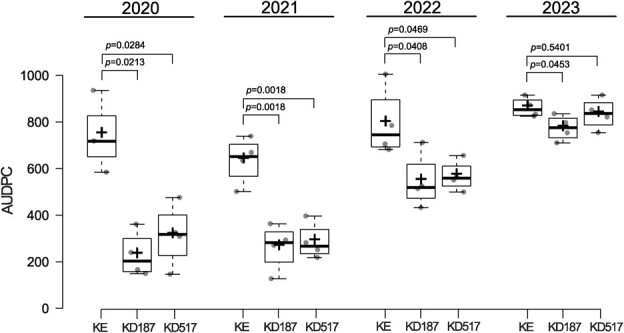
Late blight area under disease progression curve (AUDPC) shown for each genotype in the field trials for each separate year. *P* values are given for each year separately, of each mutant line compared to KE (background) in two-tailed Student’s t-tests. KE denotes the cultivar King Edward background, and KD denotes the *Stdmr6-1* mutant lines. N = 3–4.

In our previous publication with the *Stdmr6-1* mutant lines, we showed increased resistance to *P. infestans* in controlled conditions [24]. In the present study, we have collected four years of field trial data, quantifying the field resistance to complex natural *P. infestans* infestation [40]. The disease was manually scored as percentage of symptomatic foliage twice a week, and the severity was then quantified by the area under the disease progression curve (AUDPC) (Fig. 6), as this is a standard method for pathogen symptom scoring in the field and the method of quantification recommended by the International Potato Center (CIP) when investigating field resistance to the polycyclic disease late blight [41]. During the years 2020, 2021, and 2022, significantly lower disease severity was observed in both mutant lines as compared to KE (background). Generally, the disease progression curves followed a gradual increase starting mid- or end of July during these years, and the mutant lines had a slower increase of disease. In 2023, however, a significant disease reduction was observed only in the line KD187, while there was no significant decrease in disease in the line KD517 (Fig. 6). We speculate that this deviation from the trend of the previous years could be influenced by the special weather and disease pattern of the 2023 growing season. The start of the season was exceptionally dry, while the latter half was exceptionally humid. Disease onset started when the weather changed, around a week into August, and progression was quicker than any of the previous years, reaching complete infection in under two weeks. This resulted in few data points from the disease start until complete infection, revealed by the high AUDPC values for all genotypes.

Based on these results, knock-out of *StDMR6-1* emerges as a potential aid in the reduction of fungicide use, or for supporting prolonged functionality of R-genes, by mitigating infection. Similar findings of disease suppression in field conditions have been described in tomato, where severity of bacterial spot disease caused by *Xanthomonas perforans* was lower in *Sldmr6* mutants [19]. While a general decrease in susceptibility was observed in our trials, these results might not be representative of other geographical or climatic contexts.

Considering our four years of field trials, it is evident that solely targeting *StDMR6-1* will not solve the potato late blight problem without the inclusion of other integrated pest management techniques, which might involve additional knock-out targets or R-genes.

#### Yield analysis from field experiments

Yield was measured per plot and was normalized to average ton ha^−1^, accounting for the number of plants per plot each year. A major question was if the *Stdmr6-1* removal results in any yield penalty. Measurements were taken separately for the untreated plots and the plots treated with fungicide against late blight. No significant yield difference was observed in two-tailed Student’s t-tests between any of the mutant lines compared to KE (background) in 2020, 2022, or 2023 with or without fungicide treatment (Table 1). Only the mutant line KD187 had significantly lower yield in the 2021 season (Table 1). Upon analysis of the yield, no difference in tuber size distribution was observed during our potato field trials (data not shown). In field trials of the potato relative tomato (*S. lycopersicum*), a decrease of extra-large fruits was observed in *Sldmr6-1* lines, which however did not affect total marketable yield [19]. The tomato field trial was done in the humid, tropical climate of Florida, different from the temperate climate of southern Sweden. The warmer climate might have induced breakdown of SA, leading to differences in SA accumulation impacting growth, as SA has been suggested vulnerable to degradation at higher temperatures [42]. Nonetheless, tomato and potato are different crops in many respects that might experience different effects upon the deletion of this gene.

**Table 1:** Yield generated from the field trial during the consecutive years 2020–2023, given as average ton ha^−1^ for each of the genotypes in untreated and fungicide treated conditions, separately. *P* value is described for each mutant genotype, which should be interpreted as a significant difference compared to KE (background) if *P* < 0.05. Furthermore, SE and n numbers (number of plots) are provided for all genotypes.

**Year**	**Lines**	**Yield (ton∙ha** ^**−1**^ **)**	***P***	**±SE & n number**	**Lines**	**Yield (ton∙ha** ^**−1**^ **)**	***P***	**±SE & n number**
**2020**	Untreated				Fungicide treated			
	KE	36.2	NA	3.4 (n = 3)	KE	39.0	NA	1.3 (n = 4)
	KD187	30.7	0.286	3.1 (n = 4)	KD187	30.4	0.137	4.3 (n = 4)
	KD517	27.0	0.109	3.2 (n = 4)	KD517	35.4	0.220	2.2 (n = 4)
**2021**	Untreated				Fungicide treated			
	KE	10.7	NA	0.5 (n = 4)	KE	15.4	NA	1.7 (n = 4)
	KD187	7.3	0.011	0.7 (n = 4)	KD187	10.3	0.053	1.2 (n = 4)
	KD517	10.6	0.916	0.7 (n = 4)	KD517	11.8	0.163	1.5 (n = 4)
**2022**	Untreated				Fungicide treated			
	KE	31.2	NA	3.7 (n = 4)	KE	83.0	NA	32.4 (n = 2)
	KD187	40.0	0.088	1.5 (n = 4)	KD187	91.2	0.849	17.2 (n = 2)
	KD517	38.5	0.174	3.0 (n = 4)	KD517	56.3	0.559	5.0 (n = 4)
**2023**	Untreated				Fungicide treated			
	KE	30.5	NA	1.4 (n = 4)	KE	46.1	NA	3.0 (n = 4)
	KD187	30.8	0.879	1.2 (n = 4)	KD187	45.8	0.958	4.6 (n = 4)
	KD517	32.5	0.519	2.5 (n = 4)	KD517	47.0	0.848	3.1 (n = 4)

The data suggesting no yield loss reduction of *Stdmr6-*1 lines in the unsprayed plots, despite the lower disease severity, could be a cultivar-specific issue. In repeated field trials at the same site, with the same best practice cultivation, other cultivars do have such yield differences while King Edward repeatedly does not. One possible explanation is that the yield of cultivars with an earlier tuber onset can be less affected by late blight, which is the reason why earlier tuber initiation by pre-sprouted tubers has been used as a measure to decrease yield losses [43, 44]. Hence, a question could be raised about the general standard fungicide recommendations that were followed in these experiments. The recommendations do not account for cultivar or site differences, and therefore it is possible that they result in excess use of fungicides without necessarily enhancing yield for a particular cultivar, but alleviate general pathogen pressure.

Importantly in this context, after disrupting this S-gene in potato, yield of each mutant line was generally comparable to that of the background cultivar, both in fungicide-treated and untreated plots. The possibility of a yield penalty is an outstanding general question when working with S-gene mutants, as the targeted genes often have broad functions with pleiotropic effects [8]. Targeting *StDMR6-1*, and thereby regulation of the broadly active SA, certainly could be expected to be such a gene. No growth phenotype was discovered by previous above-ground studies [24], and no general yield penalty was present after four consecutive years of field trials. Passing these major hurdles greatly enhances the potential for the *Stdmr6-1* genotype to be deployed in food production.

## Conclusion

A major challenge in potato cultivation is obtaining disease resistant cultivars, with the scarcity of R-genes for numerous diseases presenting a specific obstacle. As an alternative or possibly complementary strategy, the *DMR6* (S-gene) approach has been explored in various crops. However, data from field trials and assessments of broad-spectrum stress resilience are needed to fully evaluate its potential for practical use, as well as potential detrimental side effects.

Potato *Stdmr6-1* CRISPR/Cas9 mutants have been previously described by our group with focus on increased resistance to the late blight oomycete *P. infestans* in controlled conditions [24]. Now, we show that increased resistance is generally present also in field conditions in our multiyear field test with diverse *P. infestans* populations. Interestingly, no consistent yield penalty was observed in the mutant lines. Based on the disease resistance data, it is evident that the sole use of *StDMR6-1* as an S-gene in potato breeding will not resolve the late blight problem. However, we envision that it could contribute to a reduction in fungicide application frequency and possibly extend the efficacy of resistance genes. Furthermore, there could be potential in integrating other S-gene knock-out targets to achieve an additive decrease in disease prevalence, such as the novel potato S-gene *StPM1* which is suggested to work via vacuolar degradation in contrast to SA accumulation, but also decreases susceptibility to *P. infestans* in controlled conditions, upon knock-out [9].

Notably, we also describe increased resistance to necrotrophic fungi *A. solani*, and reduced symptoms to the bacterial disease common scab. These are diseases for which no or few strong R-genes are currently known. The observed resistance to these diverse types of pathogens not only signifies a broad-spectrum resistance in the *Stdmr6-1* mutant potato but also underscores the evolutionary conservation of this S-gene across various plant species, as supported by resistance data observed in other plants. Furthermore, the increased broad resistance or tolerance observed in *Stdmr6-1* lines may extend to abiotic stressors, although this aspect requires further evaluation, especially in field conditions. Severe climate-related challenges may lie ahead, making resilient crops essential in order to sustain a more robust agricultural production system. Our field trial and other stress tolerance data further motivate the potential usefulness of field trials in other plant systems where *DMR6* has been described as an S-gene, to gain insight into the applicability across different agricultural contexts. Lastly, various tuber quality aspects were analysed, with none indicating any differences in the quality of the *Stdmr6-1* lines.

Together, these results demonstrate the prospect of *Stdmr6-1* mutants as valuable assets in future sustainable potato cultivation, which come without any apparent trade-offs.

## Materials and methods

### Plant material and *in vitro* propagation

The tetraploid potato cultivar King Edward (KE), the background genotype, along with lines of KE with *Stdmr6 1* knocked out using CRISPR/Cas9 described by Kieu *et al*. [24], were maintained *in vitro* by sub-culturing stem internodes every 3 to 4 weeks. For experimental use, apical shoots with two to three leaves were sub-cultured and left for 7 days to allow root development, before transference to experimental setups in a hydroponic system or soil. All propagation was done onto 90 × 25 mm Petri dishes containing 40 mL of Murashige and Skoog (MS) basal nutrients including vitamins (Duchefa Biochemie, M0222.0050), with 10 g/L sucrose and 4 g/L Gelrite (Duchefa Biochemie). The dishes were sealed with micropore medical sealing tape and kept in 20°C and 40 to 60 μmol/m^2^/s in a 16-hour photoperiod.

The mutant lines denoted KD78, KD187, KD517, and KD1005 were utilized for the *Alternaria solani* assay and salt stress experiments. For the PEG experiments, KD187, KD517, and KD1005 were employed, while KD517 and KD1005 were used for the drought simulation in soil. Lastly, only KD187 and KD517 were utilized in the field trial.

### *Alternaria solani* assay

A whole-plant drop inoculation assay was done using *Alternaria solani* strain 112, performed as described previously by Brouwer *et al.* [32], but using 5 weeks old plants that were inoculated with 10 μl of 100 000 spores per ml. Results were recorded by manual measuring each lesion diameter at 5 days post inoculation (dpi), and by measuring the relative pathogen biomass by the qPCR method also described by Brouwer *et al.* [32].

### Saline stress experiments

A hydroponic culture system was applied. First, rooted shoots were washed with tap water to remove any residual agar, then they were put in shared boxes containing MS basal nutrients solution (Duchefa Biochemie, 4.3 g in 5 l tap water). The boxes were kept in 20°C with a 16-hour photoperiod of 100 to 120 μmol/m^2^/s and the liquid growth medium was replaced every 2 days. After 4 days, when plants had adapted to the new environment, the liquid growth medium was exchanged to a saline medium (MS basal nutrients solution with 60 mM NaCl) to induce stress. The saline medium was renewed three times weekly. After seven additional days, the fresh weight was measured of whole individual plants, patted dry. In the control treatment plants were treated in an identical way with the exception of the addition of NaCl to the liquid medium. Samples for qPCR analysis were taken 12 hours after introduction to the saline medium. Whole plants, briefly patted dry and immediately frozen in liquid nitrogen, were sampled.

### Recovery phenotype study after osmotic stress

Apical shoots that had been rooted in MS agar in petri dishes for 1 week were transferred to a hydroponic system, which was set up in the same way as during experiments with salinity. First, plants were grown two weeks in the MS basal nutrients solution, which was renewed three times weekly. Then, the medium was replaced with a 20% polyethylene glycol (PEG)-6000 (Merck, 8 074911000) solution, diluted in tap water, to induce osmotic stress for 24 hours. Afterwards, plants and boxes were thoroughly rinsed with water to remove the PEG-6000 solution and re-cultured in the MS basal nutrients solution. After two additional weeks, during which time plants were treated identically to before the drought treatment, the fresh weight of whole plants, patted dry, was measured.

### Drought experiment in soil

Apical shoots that had been rooted in MS agar in petri dishes for one week were transferred to 0.5 L pots, each containing an equal weight of thoroughly mixed potting soil (Emmaljunga Torvmull AB, S 28022 Vittsjö, Sweden), and allowed to grow in well-watered conditions (70–80% of soil water capacity, watered daily) for two weeks in a controlled environment chamber. The chamber was kept at 20°C with 14-/10-hour light/dark cycles, light at 160 μmol∙m^−2^∙s^−1^, and humidity around 65%. After 2 weeks, watering was withdrawn, and the soil water content was monitored daily. At the same time, measurements were taken using the LI-600 (LI-COR), monitoring stomatal conductance (gsw, mol m^−2^∙s^−1^) and quantum yield of Photosystem II quantified by fluorescence (PhiPS2, or ΔF/Fm′). Stomatal conductance was measured at mid-day to early afternoon, at the peak of stomatal activity, and measurement of quantum yield followed. Three mature leaves were measured on each of six plants of each genotype and the average of each plant was used for the analysis of stomatal conductance. Two technical replicates were used for quantum yield. The area under the curve (AUC) was measured according to Simko [45] for the days number seven through ten post watering. Water was continually withheld until Day 21, at which point plants were re-watered to full water capacity and allowed to recover. The rate of recovery was observed and fresh weight was measured 2 days post recovery.

### Quantitative PCR

Extraction of mRNA was conducted on each biological replicate using the RNeasy Plant Mini Kit (Qiagen), following the manufacturer's recommendations. Subsequently, mRNA concentration and sample quality were assessed using a NanoDrop spectrophotometer (Thermo Scientific). For first-strand cDNA synthesis, 500 ng of mRNA was used. Prior to cDNA synthesis, samples were treated with DNase I (Thermo Scientific) according to the manufacturer’s protocol, with slight modifications: 1 μl of Ribolock RNase inhibitor was added to the reaction, and the termination heat treatment was adjusted to 75°C. The first-strand cDNA synthesis was conducted using the SuperScript™ III First-Strand Synthesis SuperMix for qRT-PCR by Invitrogen, following the manufacturer’s protocol, which included RNase treatment.

The qPCR template contained 10 μl SYBR Green PCR Master Mix, 0.4 μl of respective forward and reverse primer (10 μM), 7.2 μl water and 2 μl cDNA, for a total reaction volume of 20 μl. The cycling protocol started at 95°C for 3 minutes, then repeated 30 rounds of 95°C for 10 seconds followed by 60°C annealing temperature for 30 seconds. Results from the qPCR were analysed by the 2^−ΔΔCt^ method, as described by Livak and Schmittgen [46]. Primer sequences for the reference gene *StEF1α* were forward 5′ ATTGGAAACGGATATGCTCCA, and reverse 5′ TCCTTACCTGAACGCCTGTCA. Primer sequences for *StPR1* were forward 5′ GGGAGAAGCCAAACTACAACTATG and reverse 5′ ACGAGCCCGACCACAACC.

### Field trials and analysis

Field trials were conducted during the consecutive years of 2020, 2021, 2022, and 2023 in Borgeby in southern Sweden (geographic position 55.75289, 13.04872), with a rotation scheme of at least four years. The experimental design was as described in Bubolz *et al.* [40], with four randomized blocks. The only change was that the number of plants was increased from 10 to 16 per row (plot) in 2023, while the planting distance remained the same. Tubers were harvested by individual rows in the middle of September each year. Yield was measured in kg per plot, and then normalized to ton ha^−1^ with consideration of number of plants per plot each year. Late blight scoring was done as described by Bubolz *et al.* [40], from mid-June until the end of August. Late blight disease incidence based on area under the disease progress curve (AUDPC) was calculated according to Simko [45]. The whole field was sprayed against aphids with Fibro (paraffin oil) once a week, and one time with the insecticide Teppiki. For the fungicide-treated part of the trial, treatment started in late June, with application of fungicide once a week. The plots were treated with recommended doses of Revus (three times), followed by Ranman Top (two times), Infinito (two to four times), and Ranman Top (two times). All treatments were according to the manufacturer's recommendations. Fungicide treatment continued until the week before haulm killing.

The field trials were granted a permit by the Swedish Board of Agriculture (Dnr 4.6.18-01726/2020), and were performed in line with the ‘Environmental Code’ (1998;808), and the Code of Regulations of the Swedish Board of Agriculture on deliberate release of GMOs to the environment (2002:1086) and (SJVFS 2003:5).

### Common scab quantification on tubers

Potato tubers had been harvested from the trial field in the middle of September 2022, and kept in cold storage (5–8°C) in separate bags, corresponding to the separate plots in the field. After three months of cold storage, in the middle of December 2022, the tubers were washed and photographed. From the photographs, manual counting of scab lesions of each tuber (between 58 and 179 tubers from each plot) was carried out, and each plot provided one replicate given as number of lesions over total number of harvested tubers in that plot.

### Tuber quality measurements

Five tubers of similar size (approximately 70–90 mm length) were selected from each line for each quality measurement. Specific gravity was measured by a water displacement method. Each tuber was weighed in air and then weighed suspended in 4°C de-ionized water (specific gravity of 1), which revealed the tuber weight in water according to Archimedes' principle. Specific gravity was calculated for each tuber by dividing the weight in air by the sum of the weight in air minus the weight in water [38]. Furthermore, the force needed to pierce the raw tuber flesh was measured with the penetrometer Digital Fruit Hardness Tester 4 in 1 (STEP Systems GmbH), using an 8 mm diameter probe that was inserted to the halfway marking in the centre of each tuber half, and the maximum force needed was noted. As is part of standard screening in potato breeding programs, a cooking test was also performed to indicate differences in cooking ability [39]. Tubers were peeled, put in boiling water, and cooked for 20 minutes until tender, after which they were allowed to cool, cut in half, and subsequently photographed. The ratio of dry weight was measured by weighing whole fresh tubers, cutting them into quarter pieces, drying them in 60°C in an oven for 4 days, and subsequently weighing them again.

### Statistical analysis

In each respective dataset, pairwise Student's t-tests were conducted comparing each mutant genotype against the wild-type control (KE).

## Acknowledgements

This work was financially supported by Novo Nordisk Foundation (NNF19OC0057208), SLF (R-19-25-282), Formas (2020-0121; 2019-00512; 2023-01294), as well as Lyckeby Starch and KMC. We thank Mirte de Boer for assistance in maintaining potato plant material and contributing to sample preparation for qPCR.

## Author contributions

M.K, N.P.K., S.A., and E.A. jointly conceptualized the study. The investigation was carried out by M.K., N.P.K., M.A.Z., M.L., and S.R. Funding acquisition was managed by E.A., N.P.K. and M.L. The original draft was written by M.K., and E.A. contributed to writing and editing. All authors reviewed the manuscript before submission.

## Data availability

Biological material are available upon request to Erik Andreasson (Erik.Andreasson@slu.se). The numeric data used for presentation in this article are available in supplementary data S2.

## Conflict of interest statement

EA, SR, MAZ and NPK has a patent application relating to presented data SE 2430222-6.

## Supplementary Material

Web_Material_uhae130
