# Supplementary material for: CRISPR/Cas9 genome editing of potato StDMR6-1 results in plants less affected by different stress conditions
Source: Hortic Res. 2024 May 6;11(7):uhae130. doi: 10.1093/hr/uhae130 (PMC11224679; doi:10.1093/hr/uhae130)
Supplement: Web_Material_uhae130 [file web_material_uhae130.zip › Supplementary material.docx]

**Supplementary material: *CRISPR/Cas9 genome editing of potato StDMR6-1 results in plants less affected by different stress conditions***


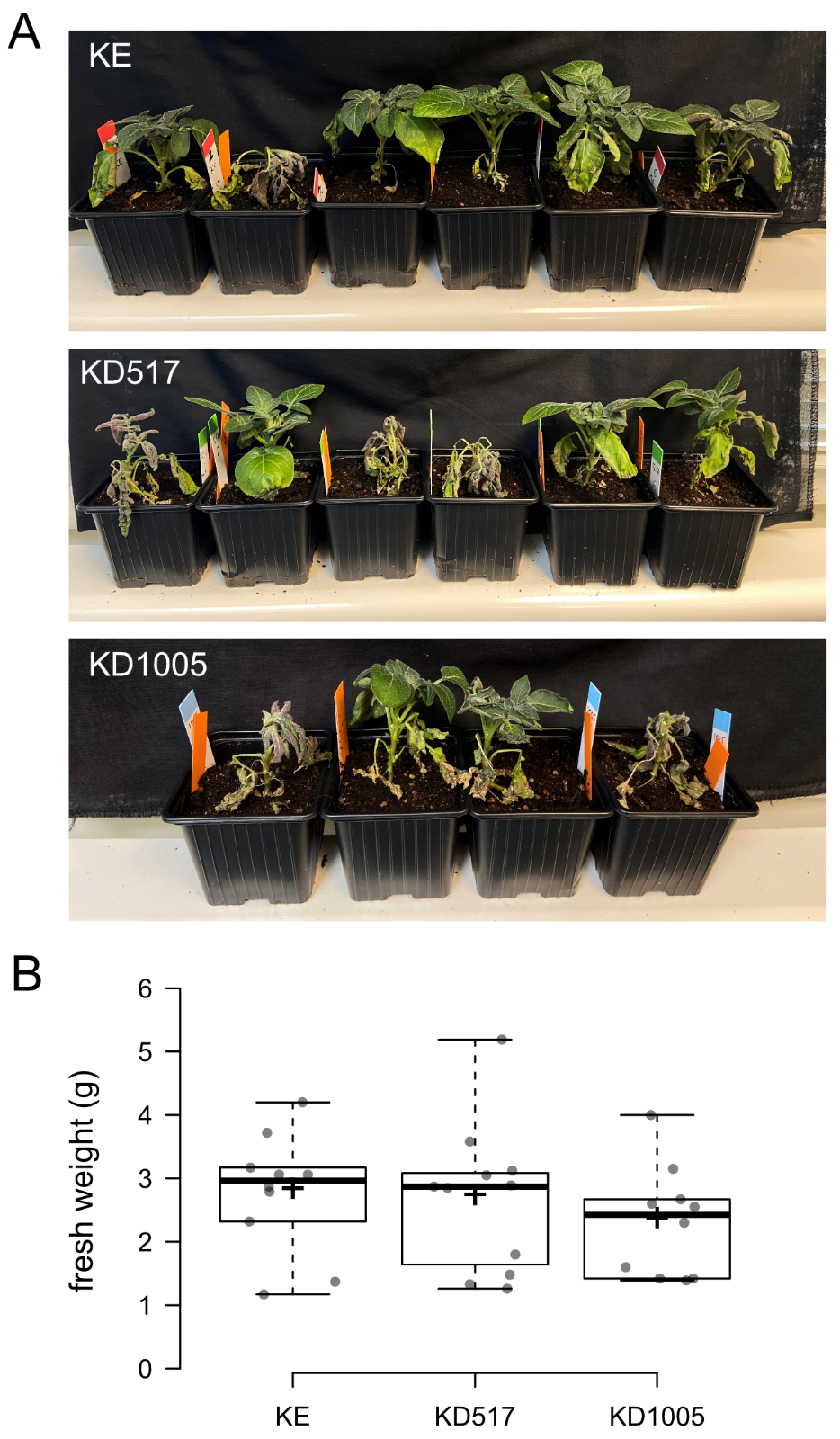


***Supplementary figure 1.*** *Recovery phenotype of Stdmr6-1 mutants two days after ending the drought. (A) Recovery of each potato line in one iteration of the experiment. (B) Fresh weight of above-ground mass from two iterations of the experiment. No significant difference was found, n=10-11.*
